# Cesium Manganese Bromide Nanocrystal Sensitizers for Broadband Vis-to-NIR Downshifting

**DOI:** 10.1021/acsenergylett.2c00311

**Published:** 2022-05-03

**Authors:** Houman Bahmani Jalali, Andrea Pianetti, Juliette Zito, Muhammad Imran, Marta Campolucci, Yurii P. Ivanov, Federico Locardi, Ivan Infante, Giorgio Divitini, Sergio Brovelli, Liberato Manna, Francesco Di Stasio

**Affiliations:** † Photonic Nanomaterials, Istituto Italiano di Tecnologia, Via Morego 30, 16163 Genova, Italy; ‡ Dipartimento di Scienza dei Materiali, Università degli Studi di Milano-Bicocca, Via R. Cozzi 55, 20125 Milano, Italy; § Department of Nanochemistry, Istituto Italiano di Tecnologia, Via Morego 30, 16163 Genova, Italy; ∥ Dipartimento di Chimica e Chimica Industriale, Università degli Studi di Genova, 16146 Genova, Italy; ⊥ Electron Spectroscopy and Nanoscopy, Istituto Italiano di Tecnologia, Via Morego 30, 16163 Genova, Italy

## Abstract

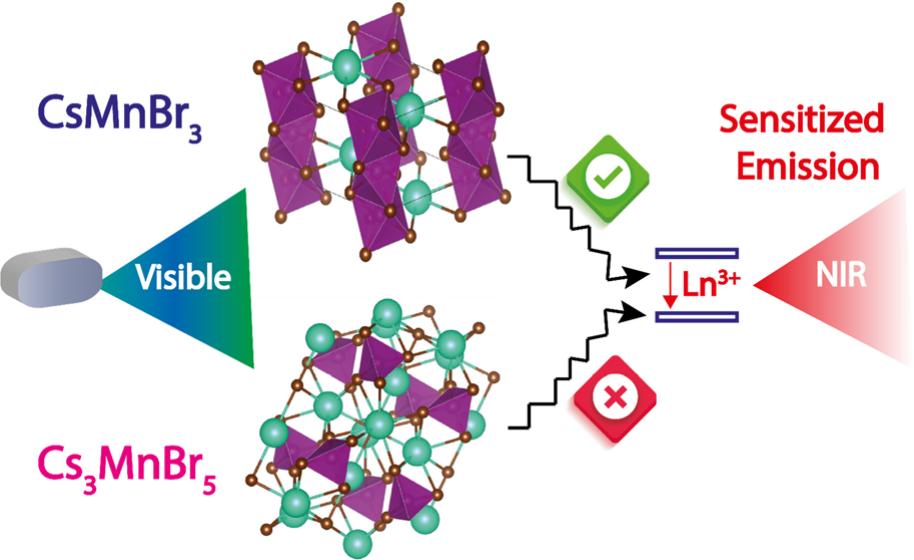

Simultaneously achieving both broad absorption and sharp emission in the near-infrared (NIR) is challenging. Coupling of an efficient absorber such as lead halide perovskites to lanthanide emissive species is a promising way to meet the demands for visible-to-NIR spectral conversion. However, lead-based perovskite sensitizers suffer from relatively narrow absorption in the visible range, poor stability, and toxicity. Herein, we introduce a downshifting configuration based on lead-free cesium manganese bromide nanocrystals acting as broad visible absorbers coupled to sharp emission in the NIR-I and NIR-II spectral regions. To achieve this, we synthesized CsMnBr_3_ and Cs_3_MnBr_5_ nanocrystals and attempted to dope them with a series of lanthanides, achieving success only with CsMnBr_3_. The correlation of the lanthanide emission to the CsMnBr_3_ visible absorption was confirmed with steady-state excitation spectra and time-resolved photoluminescence measurements, whereas the mechanism of downconversion from the CsMnBr_3_ matrix to the lanthanides was understood by density functional theory calculations. This study shows that lead-free metal halides with an appropriate phase are effective sensitizers for lanthanides and offer a route to efficient downshifting applications.

Downshifting luminescence is a single-photon process that converts absorbed high-energy photons to low-energy ones,^1^ and it is employed in a broad range of applications including solar cells,^1−3^ luminescent solar concentrators,^4^ near-infrared light emitting diodes (NIR LEDs),^5^ bioimaging,^6−8^ and biosensors.^9,10^ A common strategy for downshifting luminescence is based on a trivalent lanthanide (Ln^3+^) ion as the emission center and a sensitizer as the light absorption center. Lanthanide ions have several interesting properties as potential sensitizers, such as ladder-like electronic states and long radiative lifetimes (10 μs–10 ms),^11−14^ which can promote luminescence conversion. However, their progress as downshifters is limited since Ln^3+^ ions (e.g., Yb^3+^, Er^3+^, Tm^3+^, Nd^3+^ and Ho^3+^) have narrow absorption widths as well as very small absorption cross sections due to their electric-dipole-forbidden 4f→4f transitions.^15,16^ Although traditional semiconductor nanocrystals (NCs) such as CdSe,^17,18^ InP,^19−22^ and Ag_2_Se^23^ have a high absorption cross section and can be used as host materials, their covalently bonded rigid lattices complicate the doping process with lanthanide ions.^11,24^ Instead, halide perovskites are ideal for substitutional doping due to the softness and strong ionicity of their lattice, and additionally they offer very high absorption cross sections.^2,5,25−29^ Various reports have shown that in lead halide perovskites a downshifted luminescence in the visible and infrared spectral range can be achieved through doping with divalent cations (for instance, Cd^2+^, Mn^2+^),^30,31^ trivalent cations (Ln^3+^),^27,32,33^ or a combination thereof.^28^ Yet, the toxicity and stability issues of lead-based perovskites are a strong drive toward alternative metal halides,^34−37^ and several Pb-free double perovskites (e.g., Cs_2_AgInCl_6_,^38−40^ Cs_2_AgBiX_6_ (X = Cl, Br),^39,41^ and Cs_3_Bi_2_Br_9_^39^) have been synthesized and tested as hosts. Furthermore, most of these materials absorb only in the blue-visible range (<500 nm).^38−41^ Therefore, it is highly desirable to increase absorptivity and overall broadening of the absorption spectrum of the downshifter to the visible spectral range.

The recently reported cesium manganese bromide NCs offer several appealing properties and hold great potential as sensitizers.^42−44^ The advantages of cesium manganese bromide NCs include (i) a broad absorption spectrum covering the visible spectral range,^42,44^ (ii) high absorption coefficients (ε_544 nm_ = 83.6 M^–1^·cm^–1^ for CsMnBr_3_^42^), and (iii) significantly reduced toxicity compared to Pb-based compounds. Also, recent works have shown that the luminescence of these materials can be tuned by changing the coordination geometry of the Mn^2+^ ions since, depending on whether such coordination is tetrahedral or octahedral, the emission is either in the green or in the red spectral range, respectively.^44^ Nonetheless, it is significantly challenging to control and engineer the colloidal synthesis of cesium manganese bromide NCs due to the presence of energetically similar competing phases.^42^

Herein, we report phase-selective syntheses of colloidal Cs_3_MnBr_5_ NCs and CsMnBr_3_ NCs exhibiting green and red luminescence, respectively. We then attempt to dope both phases with various NIR-emitting lanthanide ions. Interestingly, our results show that CsMnBr_3_ can be doped successfully with Nd^3+^, Er^3+^, Tm^3+^, and Yb^3+^, while Cs_3_MnBr_5_ is inert toward all dopants due to the difficulty of lanthanides incorporation in tetrahedrally coordinated environments. Lanthanide-doped CsMnBr_3_ NCs demonstrate emission in the NIR-I (∼800–900 nm)^45^ and NIR-II (1000–1700 nm)^45^ spectral regions. These findings agree with our computational analysis on both cesium manganese bromide systems. Our study demonstrates a versatile sensitizer for downshifting luminescence of lanthanides, and it provides new opportunities for applications of lanthanide-doped nanosystems.

## Synthesis of Cs_3_MnBr_5_ and CsMnBr_3_ NCs

**Figure 1:**
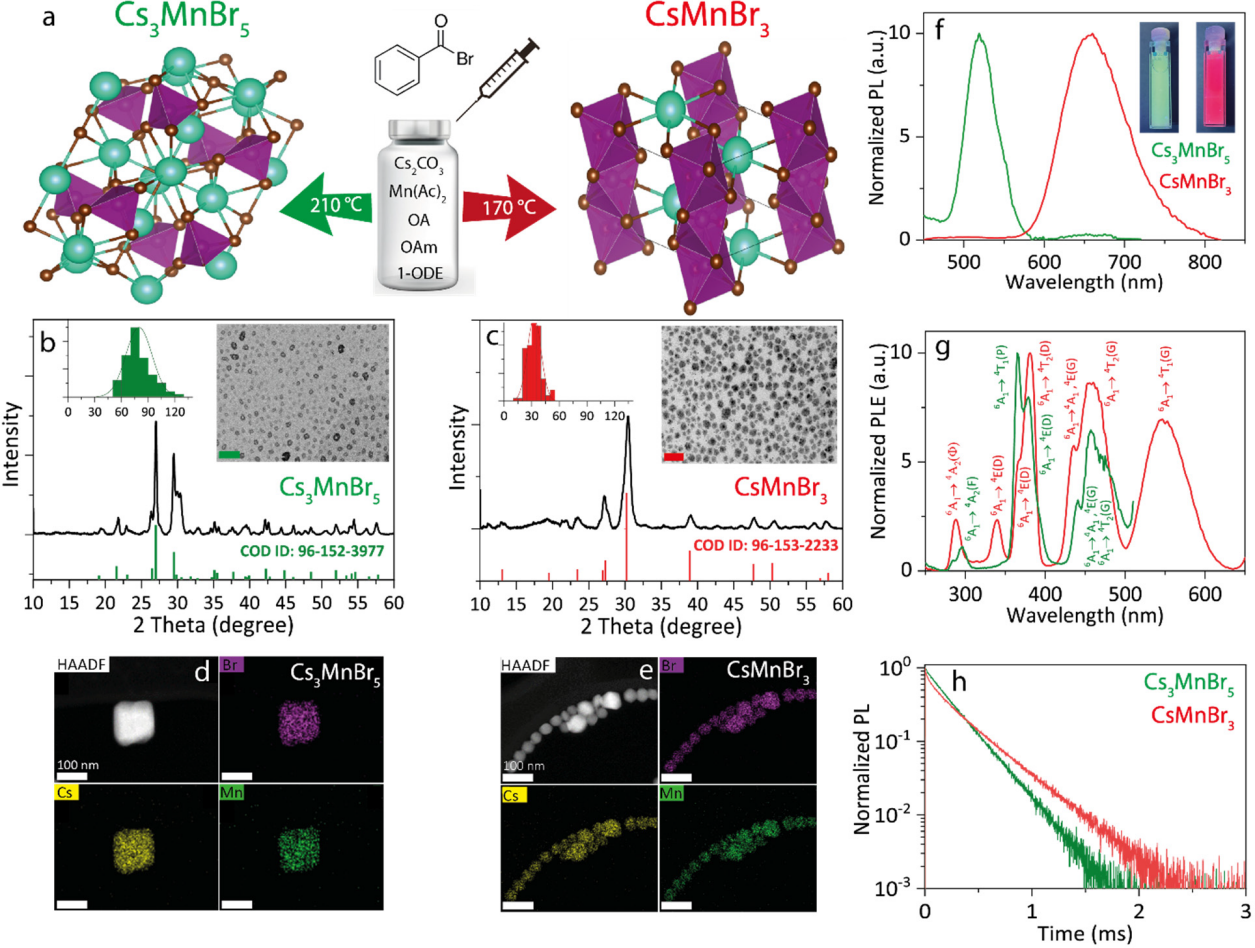
Structural and optical analyses of CsMnBr_3_ and Cs_3_MnBr_5_ NCs. (a) Schematic representation of the synthesis procedure and standard depiction (Cs = green, Mn = violet, and Br = brown) of the Cs_3_MnBr_5_ and CsMnBr_3_ structures. (b) XRD pattern and reference pattern COD ID: 96-152-3977 of the Cs_3_MnBr_5_ NCs (right inset: TEM image, scale bar = 400 nm; left inset: particle size distribution). (c) XRD pattern and reference pattern COD ID: 96-153-2233 of the CsMnBr_3_ NCs (right inset: TEM image, scale bar = 100 nm; left inset: particle size distribution). HAADF STEM image and EDS maps of (d) Cs_3_MnBr_5_ and (e) CsMnBr_3_ NCs. (f) Photoluminescence (PL) spectra of the Cs_3_MnBr_5_ (green, λ_exc_ = 380 nm) and CsMnBr_3_ (red, λ_exc_ = 380 nm) NCs dispersed in toluene. Inset: Photographs of the Cs_3_MnBr_5_ and CsMnBr_3_ NC solutions under UV light. (g) Photoluminescence excitation (PLE) spectra of the Cs_3_MnBr_5_ (green, λ_em_ = 522 nm) and CsMnBr_3_ (red, λ_em_ = 661 nm) NCs dispersed in tolouene. (h) PL time decay of Cs_3_MnBr_5_ (green, λ_em_ = 522 nm) and CsMnBr_3_ (red, λ_em_ = 661 nm) NCs dispersed in toluene.

As mentioned earlier, the synthesis of phase-pure cesium manganese bromide-based NCs is more challenging than that of classical lead halide perovskite NCs due to the presence of different competing phases that can be easily formed in the CsBr–MnBr_2_ phase diagram, such as CsMnBr_3_, Cs_3_MnBr_5_, and Cs_2_MnBr_4_.^46^ In a recent study, CsMnBr_3_ and Cs_3_MnBr_5_ NCs were synthesized in a Schlenk line system using the highly reactive trimethylbromosilane as the bromide source.^44^ As a safer synthesis route, we prepared here Cs_3_MnBr_5_ and CsMnBr_3_ NCs using a modified version of our previously developed benzoyl halide-based synthesis approach, which enables independent tunability of the concentration of metal cations, halide ions, and surfactants.^47−49^ Briefly, NCs were synthesized by injecting benzoyl bromide into a solution of cesium and manganese oleates in the presence of oleylamine (see scheme in Figure 1a). We found that CsMnBr_3_ and Cs_3_MnBr_5_ NCs can be separately prepared, each with high phase purity, under optimized sets of reaction conditions (precursors with tailored ratios, reaction temperature, and time; see the details in the Experimental Methods). X-ray diffraction (XRD) analysis indicates that CsMnBr_3_ NCs have a hexagonal crystal structure (*P*6_3_/*mmc* space group) formed by chains of face-sharing [MnBr_6_] octahedra that are charge-balanced by cesium ions along the *c*-axis (Figure 1a). The Cs_3_MnBr_5_ NCs has instead a tetragonal crystal structure formed by isolated [MnBr_4_] tetrahedra (each Mn^2+^ ion is bound in a tetrahedral configuration to four Br^–^ ions) and stabilized by cesium ions (*I*4/*mcm* space group, Figure 1a). These results are consistent with existing literature on bulk CsMnBr_3_^50,51^ and Cs_3_MnBr_5_^52^ crystals. According to transmission electron microscopy (TEM) analysis, the NCs has a mean size of 78 ± 14 nm (Figure 1b, inset) and 33 ± 7 nm (Figure 1c, inset) for Cs_3_MnBr_5_ and CsMnBr_3_ NCs, respectively. The much larger size of the Cs_3_MnBr_5_ NCs can be attributed to the higher injection temperature (210 °C) required in their synthesis compared to the CsMnBr_3_ NCs case (170 °C). HAADF STEM images in combination with EDS mapping confirm compatible compositions for Cs_3_MnBr_5_ (Figure 1d) and CsMnBr_3_ NCs (Figure 1e and Table S1).

The optical properties and electronic structure of manganese halides are due to electronic transitions localized in the [MnBr_*x*_] (*x* = 4, 6) polyhedra that are dominated by d-d transitions within the Mn cations mixed with some orbital contribution from the nearby 4p of the Br ions. These excitations are typically spin and parity forbidden;^53−56^ nevertheless, exchange coupling as well as spin–orbit coupling are responsible for the relaxation of spin selection rules in antiferromagnetic manganese halides.^56^ These optical properties can be tuned by changing the coordination geometry around the Mn^2+^ ions and the Mn–Mn distance.^57,58^ In particular, tetrahedrally coordinated Mn^2+^ exhibits green emission,^55^ while octahedrally coordinated Mn^2+^ exhibits red emission.^59,60^ We studied the steady-state optical properties in colloidal dispersions to reconfirm the presence of Cs_3_MnBr_5_ and CsMnBr_3_ NCs. Cs_3_MnBr_5_ NCs features green emission centered at 522 nm, while the CsMnBr_3_ NCs shows red emission centered at 661 nm (Figure 1f), and both phases show photoluminescence quantum yields (PLQYs) in the range of 33 ± 4%, which decreases to 8 ± 2% after storage in the ambient air for 10 days (relative humidity ∼40%). Importantly, the Cs_3_MnBr_5_ NC solution shows only negligible emission in the red spectral region compared to the literature. In fact, the only reported Cs_3_MnBr_5_ NCs’ photoluminescence to date had two intense emissions around 520 and 660 nm,^44^ indicating the actual presence of both Cs_3_MnBr_5_ and CsMnBr phases in that sample. The optical absorption spectra for the Cs_3_MnBr_5_ and CsMnBr_3_ NCs prepared by us are reported in Figure S1, while Figure 1g displays the photoluminescence excitation (PLE) spectra, in which the d-d transition of the Mn^2+^ ion in d^5^ configuration and different excitation states funnel the excitation to the same transition (^6^A_1_→^4^T_1_(G)) for both tetrahedral Cs_3_MnBr_5_^55^ and octahedral CsMnBr_3_^59,60^ NCs (Figure 1g). The PL time decays of CsMnBr_3_ and Cs_3_MnBr_5_ NC solutions reveal a single exponential kinetics at room temperature. The Cs_3_MnBr_5_ NCs, emitting at 522 nm, decay faster (τ = 170 μs) than the CsMnBr_3_ NCs (τ = 235 μs), the latter emitting at 661 nm, which is in agreement with the literature (Figure 1h, Table S2).^44^ Interestingly, no excitation-dependent PL decay lifetime has been observed for CsMnBr_3_ and Cs_3_MnBr_5_ NCs (excitation at 532 nm vs 355 nm).

## Vis-to-NIR Downshifting Using Ln^3+^-Doped CsMnBr_3_ NCs

**Figure 2:**
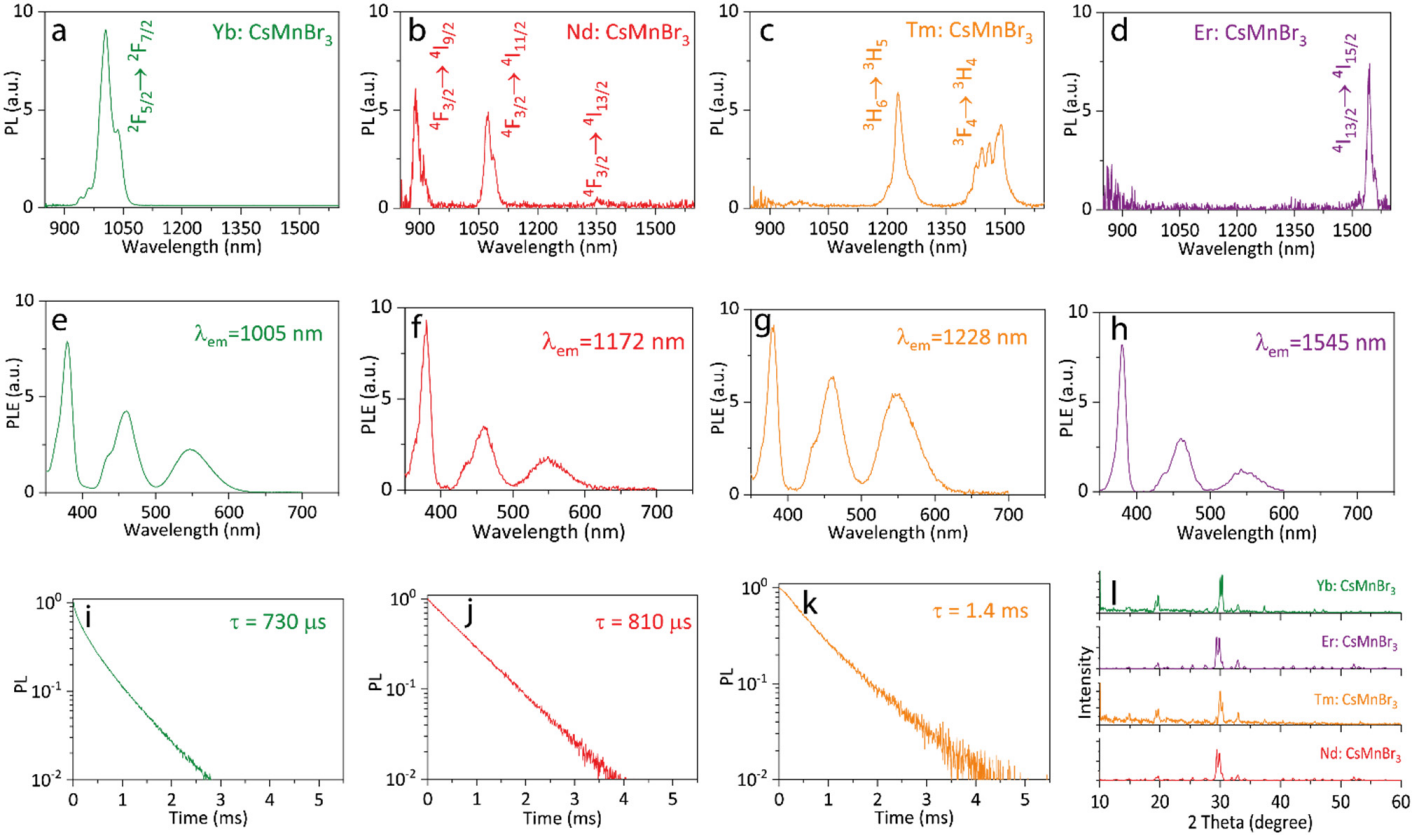
Vis-to-NIR downshifting using Ln^3+^-doped CsMnBr_3_ NCs. (a) Yb-doped, (b) Nd-doped, (c) Tm-doped, and (d) Er-doped CsMnBr_3_ NCs (λ_exc_ = 550 nm). PLE spectra of (e) Yb-doped (λ_em_ = 1005 nm), (f) Nd-doped (λ_em_ = 1172 nm), (g) Tm-doped (λ_em_ = 1228 nm), and (h) Er-doped (λ_em_ = 1545 nm) CsMnBr_3_ NCs. PL decay curves of (i) Yb-doped (λ_det_ = 990 nm), (j) Nd-doped (λ_det_ = 895 nm), and (k) Tm-doped (λ_det_ = 1485 nm) CsMnBr_3_ NCs (λ_exc_= 355 nm). (l) XRD patterns of the CsMnBr_3_ NCs doped with Yb, Er, Tm, and Nd ions.

Sensitizers hosting different types of lanthanides and efficiently absorbing in the visible spectral range are highly desirable for downshifting applications.^19,61^ This motivated us to investigate the performance of both CsMnBr_3_ and Cs_3_MnBr_5_ NCs as sensitizers. For that, we attempted doping with different lanthanide dopants, including Yb^3+^, Nd^3+^, Tm^3+^, and Er^3+^, via a facile synthesis route (see Experimental Methods), on both NC systems. Energy-dispersive X-ray spectroscopy (EDS) revealed unsuccessful doping of the Cs_3_MnBr_5_ NCs (Figure S2). The apparently ineffective doping of Cs_3_MnBr_5_ NCs with lanthanides might stem from the difficulty to incorporate lanthanides (preferring CN ≥ 6^62−64^) in a tetrahedrally coordinated environment or by the lack of a favorable energy alignment between the dopant and the Mn matrix (*vide infra*).^11^ On the other hand, CsMnBr_3_ NCs were successfully doped by Yb^3+^, Nd^3+^, Tm^3+^, and Er^3+^ (Figures S3–S6). However, for the case of CsMnBr_3_, two Ln^3+^ ions can, in principle, substitute three Mn^2+^ ions, generating a cation vacancy (V_Mn_),^65^ as was reported for CsMnCl_3_^66,67^ and CsPbX_3_.^2,3,26,68−72^ The higher content of Nd (1.22 at.%) compared to Yb (1.19 at.%), Er (1.02 at.%), and Tm (0.89 at.%) that we could introduce in the NCs can be explained by the lower ionic radius mismatch between Mn^2+^ (97 ppm^73^) and Nd^3+^ (98.3 ppm^74^) compared to Er^3+^ (89 ppm^74^), Tm^3+^ (87 ppm^75^), and Yb^3+^ (86 ppm^76^). In addition, XRD patterns of lanthanide-doped CsMnBr_3_ NCs indicate no extra diffraction peaks nor any notable shift compared to undoped CsMnBr_3_ NCs (Figure 2l) due to the low amount of lanthanides (<1.5 at.%) that could be introduced in the lattice, as reported for Er-doped^27^ and Yb-doped^26^ CsPbCl_3_ NCs.

We observed NIR emission features via excitation in the visible spectral range (550 nm) for CsMnBr_3_ NCs doped with Yb^3+^, Nd^3+^, Tm^3+^, and Er^3+^ (Figure 2a–d) having NIR PLQYs in the range of 0.24–1.1% (see Table S4). Furthermore, the lanthanide-doped CsMnBr_3_ NCs have the same PLE profile as the undoped CsMnBr_3_ NCs toward all types of dopants (Figure 2e–h). This suggests that lanthanide NIR emission is triggered by excitation of the host CsMnBr_3_ NCs. This phenomenon is in agreement with the efficient energy transfer from Mn^2+^ commonly observed in lanthanide-doped bulk CsMnBr_3_,^56,77,78^ CsMnCl_3_,^56,79^ and CsMnI_3_.^56,77^ In our case, since the NCs are small and the doping level is relatively higher (around 1 part per 80, 84, 100, and 112 for Nd,- Yb-, Er-, and Tm-doped CsMnBr_3_, respectively) than the one reported for doped CsMnBr_3_ (1 part per 1000 for Nd:CsMnBr_3_^80^ and 1 part per 500 for Er:CsMnBr_3_^77^), energy transfer does not require migration of excitation among all Mn sites. For this reason, the introduction of lanthanides quenches completely the emission from the Mn-centered d-d transition of CsMnBr_3_ at 661 nm. The near-infrared PL time decays show a single-exponential decay with a lifetime of 810, 730, and 1400 μs for Nd:CsMnBr_3_, Yb:CsMnBr_3_, and Tm:CsMnBr_3_, respectively (Figure 2i–k, Table S3), which also shows that there is no interdoping effect.

## Computational Analysis of Ln^3+^-Doped CsMnBr_3_ and Cs_3_MnBr_5_ NCs

**Figure 3:**
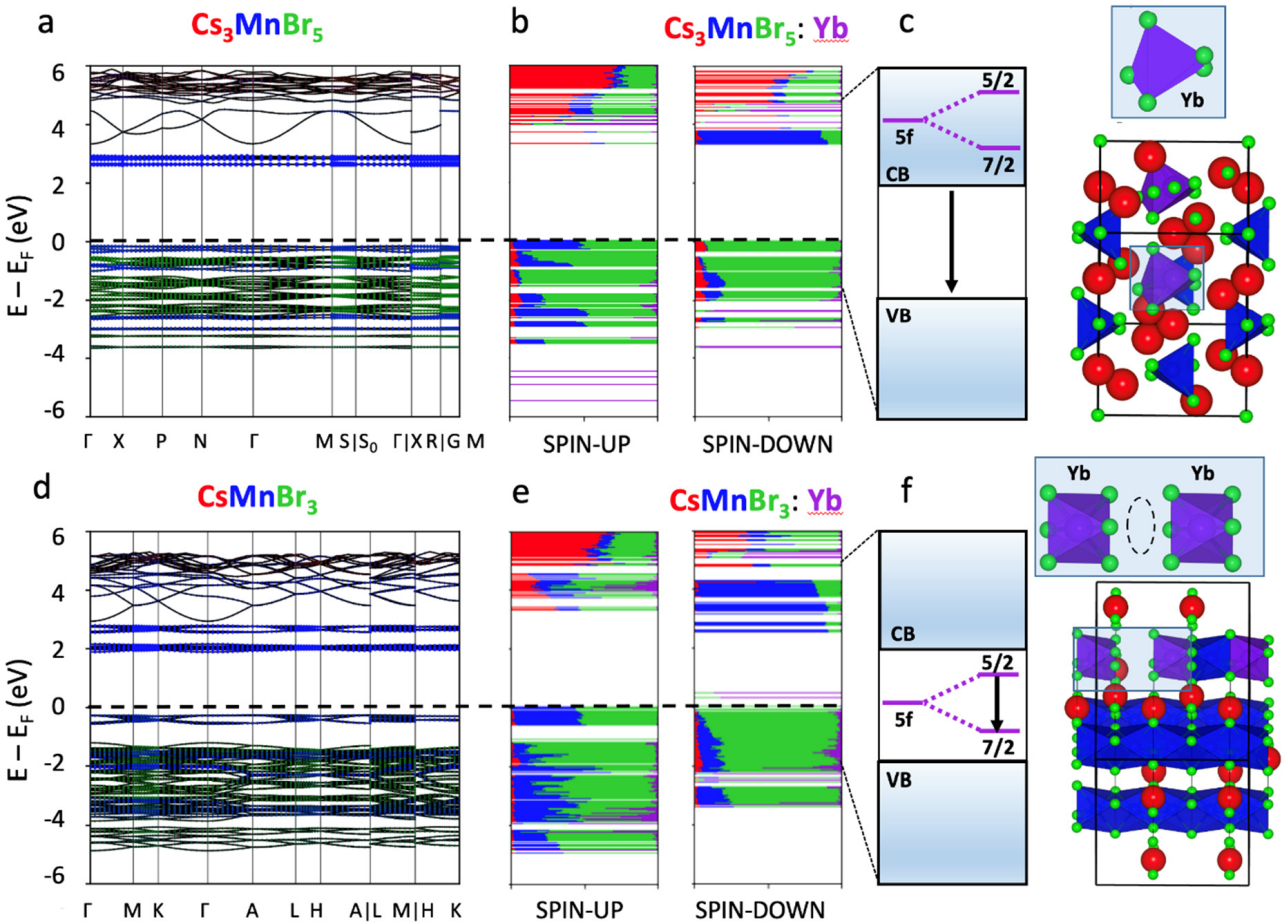
Density functional theory analyses of undoped and doped Cs_3_MnBr_5_ and CsMnBr_3_ systems. (a) Element projected band structure of the relaxed Cs_3_MnBr_5_ unit cell calculated at the DFT/PBE level of theory. (b) Electronic structure of the Yb-doped Cs_3_MnBr_5_ 2×2×2 supercell (shown on the right) computed at the Γ point at the DFT/PBE level of theory. Each orbital is represented in real space and decomposed according to each atom type. (c) Scheme of the expected position for Yb 5f_5/2_ and 5f_7/2_ orbitals upon spin–orbit mixing. (d), (e), and (f) are the same as (a), (b), and (c), respectively, but for the CsMnBr_3_ system.

To disentangle the mechanism of emission of lanthanide-doped CsMnBr_3_ and Cs_3_MnBr_5_ NCs, we carried out DFT calculations. First, the band structures of both undoped CsMnBr_3_ and Cs_3_MnBr_5_ systems were computed at the DFT/PBE level (Figure 3a,d). In these band structures, the flat conduction and valence band edges are dominated by localized Mn half-filled d orbitals, which confirms that the emission arises from the d-d transition of Mn^2+^ ion in d^5^ configuration (see Figure 1d,e). Our calculations also indicate that both systems slightly favor an antiferromagnetic behavior (see the “Density Functional Theory calculations” paragraph in the Experimental Methods). For the doped systems, we decided to analyze the Yb doping since Yb presents only one unpaired electron that gives origin to one emission line (^2^F_5/2_→^2^F_7/2_) upon spin–orbit mixing and greatly facilitates the interpretation of the results. To improve the convergence of our DFT calculations, we considered spin-free calculations, i.e., no spin–orbit coupling: we doubled the size of the unit cell to perform the computation only in the Γ point and assumed a purely ferromagnetic behavior, with all unpaired electrons in Mn and Yb occupying the spin-up orbitals. We consider this latter approximation as valid due to the very small energetic difference from the purely antiferromagnetic systems. We do, however, warn the reader that based on the above approximations and considering that DFT has limitations in describing f-orbitals with high precision, we aim to acquire only a purely qualitative description of the doped systems.

Starting with the density of states of the Cs_3_MnBr_5_:Yb system (Figure 3b), composed of disconnected tetrahedra, we can notice that even in the case when Yb is incorporated in Cs_3_MnBr_5_, the occupied f-orbitals of Yb lie very deep in the valence band, whereas the only unoccupied f orbital (spin-down) is found at energies higher than the Mn d-orbitals. In Figure 3c, we show schematically that even if spin–orbit coupling would be considered, the Yb orbitals would probably lie above the conduction band, thus possibly preventing any energy transfer from the Mn d-orbitals, which we know absorb light from the PLE spectra of the doped systems. On the other hand, the CsMnBr_3_ system presents a different electronic structure. The 1D connectivity among Mn and Yb octahedra moves the unoccupied f-orbital (spin-down) deep inside the band gap of the material, as shown in Figure 3e. The composition (in terms of atomic orbital contribution) of the unoccupied molecular orbitals (spin-down) localized on the Yb^3+^ dopant(s) is provided in Tables S5–S7. Although the exact energetic position inside the gap is probably not well reproduced by DFT, we can safely assume that even after spin–orbit mixing, both 5f_5/2_ and 5f_7/2_ orbitals would still lie in the band gap, allowing emission from the dopant (Figure 3f). Additionally, we can also observe that the f-orbital is mixed with the 4p orbitals of Br, which are directly connected to the nearby Mn ions. This means that the conversion efficiency from Mn to Yb could be facilitated by electron–phonon coupling. A similar mechanism can be expected also from the other dopants.

In summary, we have introduced an optimized synthesis of CsMnBr_3_ and Cs_3_MnBr_5_ NCs. Importantly, only CsMnBr_3_ NCs could host different types of lanthanide ions and sensitize them via visible excitation, which was shown in our DFT calculations as well. As a result, sharp emissions at 890 and 1075 nm from Nd^3+^, 1005 nm from Yb^3+^, 1226 and 1489 nm from Tm^3+^, and 1544 nm from Er^3+^ were detected upon visible excitation of the CsMnBr_3_ NCs matrix. This work provides a lead-free material as an efficient sensitizer, which can lead to development and design of visible-to-NIR downshifters.

## Experimental Methods

### Materials

Cesium carbonate (Cs_2_(CO_3_), 99%), manganese(II) acetate (Mn(Ac)_2_, 98%), benzoyl bromide (C_6_H_5_COBr, 97%), oleic acid (OA, 90%), oleylamine (OAm, 98%), 1-octadecene (1-ODE, 90%), erbium(III) acetate hydrate (Er(Ac)_3_·H_2_O, 99.9%), ytterbium(III) acetate tetrahydrate (Yb(Ac)_3_·4H_2_O, 99.9%), thulium(III) acetate hydrate (Tm(Ac_)3_·H_2_O, 99.9%), neodymium(III) acetate hydrate (Nd(Ac)_3_·H_2_O, 99.9%), ethyl acetate (99.9%), and toluene (99.7%) were purchased from Sigma-Aldrich and used without further purification.

### Synthesis of CsMnBr_3_ NCs

Cs_2_(CO_3_) (65 mg), Mn(Ac)_2_ (70 mg), OAm (1 mL), and OA (1 mL) were mixed in 1-ODE (2 mL). The solution was degassed at room temperature for 30 min and then filled with nitrogen. The solution was heated to 170 °C to form a clear mixture. Then, benzoyl bromide (450 μL in 0.5 mL toluene) was swiftly injected into the solution, and the reaction was quenched within 30 s using an ice–water bath. The crude solution was centrifuged at 4000 rpm for 5 min, and the precipitate was redispersed in toluene. The same washing procedure was repeated for three times.

### Synthesis of Cs_3_MnBr_5_ NCs

Cs_2_(CO_3_) (90 mg), Mn(Ac)_2_ (70 mg), OAm (1 mL), and OA (1 mL) were mixed in 1-ODE (2 mL). The solution was degassed at room temperature for 30 min and then filled with nitrogen. The solution was heated to 210 °C to form a clear mixture. Then, benzoyl bromide (225 μL in 0.5 mL toluene) was swiftly injected into the solution, and the reaction was quenched within 30 s using an ice–water bath. The crude solution was centrifuged at 4000 rpm for 5 min, and the precipitate was redispersed in toluene. The same washing procedure was repeated three times.

### Ln^3+^ Doping of CsMnBr_3_ and Cs_3_MnBr_5_ NCs

Er(Ac)_3_·H_2_O (41 mg), Yb(Ac)_3_·4H_2_O (60 mg), Tm(Ac)_3_·H_2_O (42 mg), and Nd(Ac)_3_·H_2_O (40 mg) were introduced into the synthesis batch of CsMnBr_3_ and Cs_3_MnBr_5_ NCs.

### X-ray Diffraction (XRD) Characterization

XRD analysis was carried on a PANanalytical Empyrean X-ray diffractometer, equipped with a 1.8 kW Cu Kα ceramic X-ray tube and a PIXcel3D 2×2 area detector, operating at 45 kV and 40 mA. A concentrated NC solution was drop-cast onto silicon zero-diffraction single-crystal substrate for the analysis, which was collected under ambient conditions using parallel beam geometry and symmetric reflection mode. The HighScore 4.1 software from PANalytical was used for data analysis.

### Transmission Electron Microscopy (TEM) Characterization

TEM analysis was performed on a JEOL-1100 transmission electron microscope operating at an acceleration voltage of 100 kV. The dilute solutions of NCs were drop-cast onto carbon-coated copper grids. The TEM images were processed by the ImageJ software (https://imagej.nih.gov/ij/) for particle size determination. Scanning transmission electron microscopy (STEM) images were acquired on a ThermoFisher Spectra instrument operated at 300 kV using the high-angle annular dark field (HAADF) signal. EDS maps were acquired on a Dual-X setup with a total acquisition angle of 1.76 sr and processed with Velox.

### UV–Vis Absorption

The UV–vis absorption spectra were recorded using a Varian Cary 300 UV–vis absorption spectrophotometer. Diluted NC solutions were dispersed in toluene in quartz cuvettes with a path length of 1 cm.

### Steady-State Optical Analyses

The PLE, visible PL and NIR PL spectra were collected via an Edinburgh FLS900 fluorescence spectrometer equipped with a Xe lamp and a monochromator for steady-state PL excitation.

### Photoluminescence Quantum yield Measurements

An Edinburgh FLS900 fluorescence spectrometer equipped with a Xe lamp, PMT-900 detector, PMT-1700 detector, and calibrated integrating sphere (N-M01) was used for PLQY measurement. Undoped samples were excited at 380 nm for the visible PLQY measurements, and doped samples were excited at 550 nm for the NIR PLQY measurements. The PLQY values were calculated by Flouracle software.

### Near-Infrared Photoluminescence Time Decay

For transient PL measurements, the samples were excited using a Laser-export Co. Ltd., frequency-tripled, pulsed Nd:YAG laser at 355 nm (3.49 eV) with modulable repetition rate (from 1 kHz down to 150 Hz) and detected using a Oriel Instrument Cornerstone 1/4 m monochromator coupled with a Hamamatsu UV–vis photomultiplier and a Hamamatsu R5509 NIR photomultiplier tube cooled at liquid nitrogen temperature with a Products for Research, Inc. PC176TSCE005 cooling chamber.

### Density Functional Theory Calculations

The band structure calculations of the undoped systems were performed using the VASP 5.4 package^81^ at the DFT level using the PBE exchange–correlation functional^82^ and without further inclusion of the spin–orbit coupling. We considered the tetragonal space group (SG) No. 140 for Cs_3_MnBr_5_ and the hexagonal SG No. 194 for CsMnBr_3_ using, respectively, a 4×4×4 and a 6×6×6 k mesh grid for the Brillouin zone integration. All atomic positions and lattice parameters were relaxed until forces were <0.001 hartree/Å. We used a kinetic energy cutoff of 400 eV. To assess the impact of the magnetic behavior, we compared the stability of the pure ferromagnetic (five unpaired electrons on each Mn, spin-up) and pure antiferromagnetic (five unpaired electrons on each Mn, alternating spin-up and spin-down for adjacent Mn) configurations in both systems after structural relaxation. In order to investigate the effects of Yb-doping in both systems, we carried out atomistic simulations at the Γ point of the corresponding 2×2×2 supercells. In detail, we prepared a 2×2×2 Cs_3_MnBr_5_ supercell, replacing one Mn^2+^ with one Yb^3+^, and added a Br^–^ ion to the corresponding tetrahedron in order to ensure the charge balance of our computational model (see Figure 3, upper right panel). Similarly, we built a 2×2×2 CsMnBr_3_ supercell and replaced three neighboring (edge-connected) Mn^2+^ respectively by an Yb^3+^, a vacancy, and a Yb^3+^ (see Figure 3, lower right panel). The structural relaxation and electronic structure calculation of such supercells were accomplished at the DFT/PBE level using a double-ζ basis set plus polarization functions on all atoms^83^ as implemented in the CP2K 8.1 code.^84^ Scalar relativistic effects have been incorporated as effective core potentials. Here, only the purely ferromagnetic (five unpaired electrons on each Mn, spin-up, and one unpaired electron on each Yb, spin-up) behavior was modeled.

## Supporting Information Available

EDS mapping data, UV–vis absorption and EDX spectra, PL decay lifetime values, and composition of the different orbitals of Yb-doped CsMnBr_3_ and Cs_3_MnBr_5_ NCs, including Tables S1–S7 and Figures S1–S6 (PDF)
