# Supplementary material for: Cesium Manganese Bromide Nanocrystal Sensitizers for Broadband Vis-to-NIR Downshifting
Source: ACS Energy Lett. 2022 May 3;7(5):1850–8. doi: 10.1021/acsenergylett.2c00311 (PMC9112327; doi:10.1021/acsenergylett.2c00311)
Supplement: Supplementary file 1 — nz2c00311_si_001.pdf [file nz2c00311_si_001.pdf]

SUPPORTING INFORMATION

Cesium Manganese Bromide Nanocrystal Sensitizers for Broadband Vis-to-NIR Downshifting

Houman Bahmani Jalali[a], Andrea Pianetti[b], Juliette Zito[c,d], Muhammad Imran[c], Marta Campolucci[d], Yurii P. Ivanov[e], Federico Locardi[d], Ivan Infante[c], Giorgio Divitini[e], Sergio Brovelli[b], Liberato Manna[c], and Francesco Di Stasio [a]**

[a] Photonic Nanomaterials group, Istituto Italiano di Tecnologia, Via Morego 30, 16163 Genova, Italy

[b] Dipartimento di Scienza dei Materiali, Università degli Studi di Milano-Bicocca, Via R. Cozzi 55, 20125 Milano, Italy

[c] Department of Nanochemistry, Istituto Italiano di Tecnologia, Via Morego 30, 16163 Genova, Italy

[d] Dipartimento di Chimica e Chimica Industriale, Università degli Studi di Genova, 16146 Genova, Italy

[e] Electron Spectroscopy and Nanoscopy, Istituto Italiano di Tecnologia, Via Morego 30, 16163 Genova, Italy

Table S1. HAADF STEM EDS mapping data of the $CsMnBr_3$ and Cs_3MnBr_5 NCs.

Element	Cs_3MnBr_5	$CsMnBr_3$
**Cs (at. %)**	31 ± 3	18 ± 2
**Mn (at. %)**	13 ± 1	19 ± 2
**Br (at. %)**	57 ± 4	63 ± 3

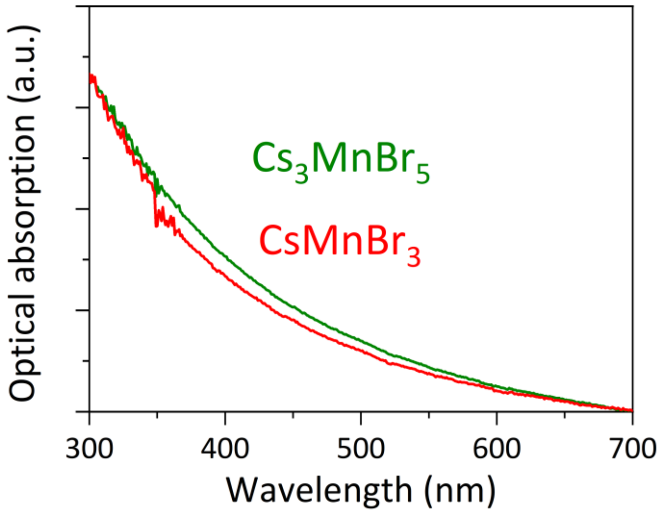

Figure S1. UV/Visible absorption of the Cs_3MnBr_5 (green) and $CsMnBr_3$ (red) NCs dispersed in toluene.

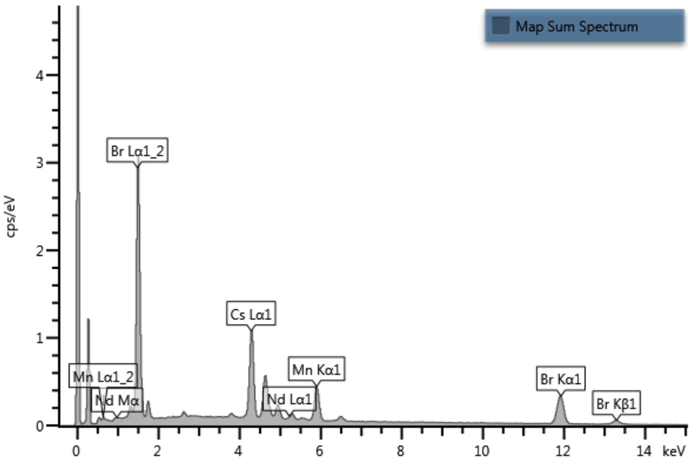

Figure S2. EDX spectrum recording from Nd doped Cs_3MnBr_5 NCs, showing the existence of Cs, Mn and Br elemental signals

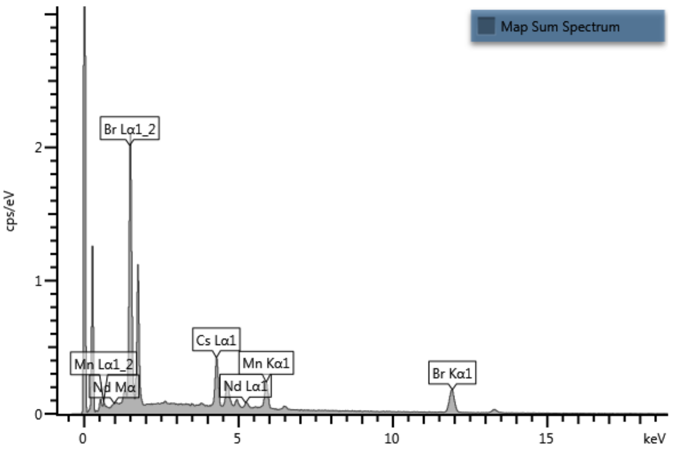

Figure S3. EDX spectrum recording from Nd doped $CsMnBr_3$ NCs, showing the existence of Cs, Mn, Br and Nd elemental signals

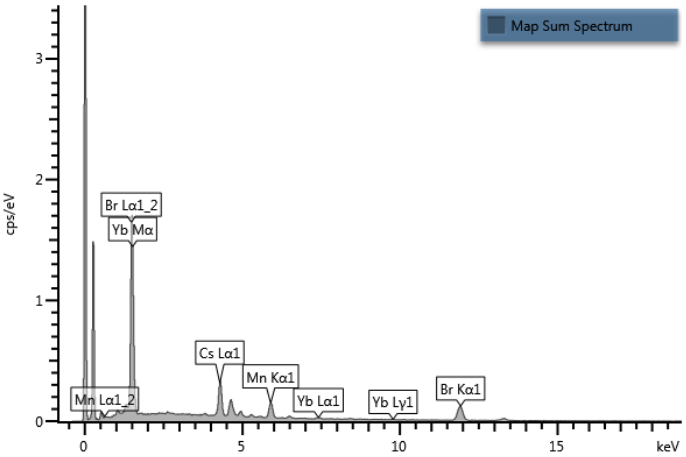

Figure S4. EDX spectrum recording from Yb doped $CsMnBr_3$ NCs, showing the existence of Cs, Mn, Br and Yb elemental signals

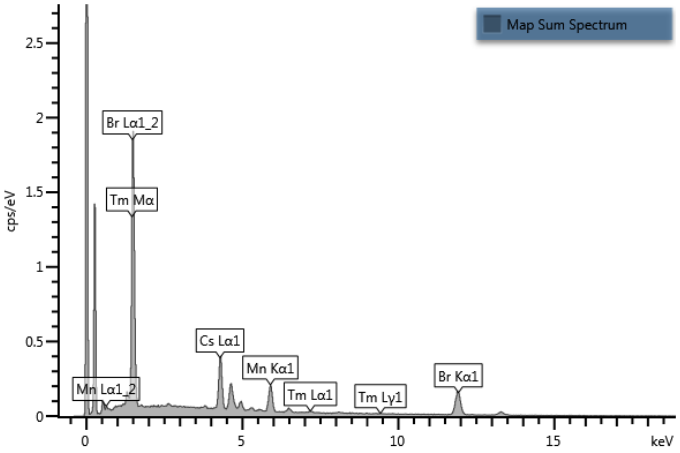

Figure S5. EDX spectrum recording from Tm doped $CsMnBr_3$ NCs, showing the existence of Cs, Mn, Br and Tm elemental signals

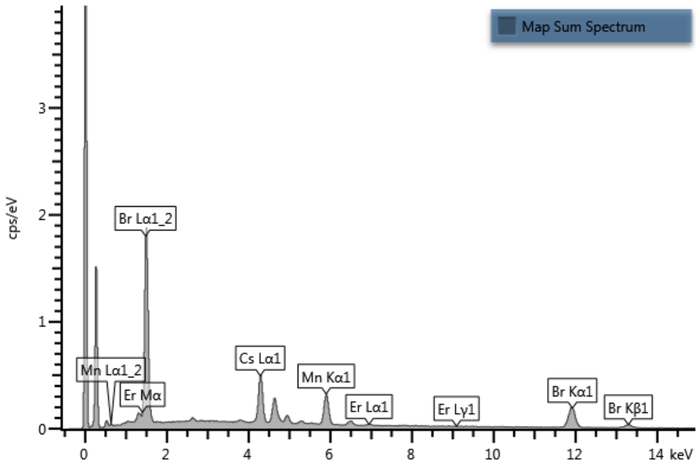

Figure S6. EDX spectrum recording from Er doped $CsMnBr_3$ NCs, showing the existence of Cs, Mn, Br and Er elemental signals

Table S*2*. Visible PL decay lifetimes of the $CsMnBr_3$ and Cs_3MnBr_5 NCs.

Sample	Emission wavelength (nm)	Lifetime (τ)
$CsMnBr_3$ NCs	661	235 µs
Cs_3MnBr_5 NCs	522	170 µs

Table S3. NIR PL decay lifetimes of the Ln^{3+} doped $CsMnBr_3$ NCs.

Dopant	Emission wavelength (nm)	Lifetime (τ)
Nd^{3+} doped $CsMnBr_3$ NCs	895	810 µs
Tm^{3+} doped $CsMnBr_3$ NCs	1230	1.4 ms
Yb^{3+} doped $CsMnBr_3$ NCs	990	730 µs

Table S4. NIR PLQY of the Ln^{3+} doped $CsMnBr_3$ NCs.

Dopant	NIR PLQY (%)
Nd^{3+} doped $CsMnBr_3$ NCs	0.41
Tm^{3+} doped $CsMnBr_3$ NCs	0.63
Yb^{3+} doped $CsMnBr_3$ NCs	1.1
Er^{3+} doped $CsMnBr_3$ NCs	0.24

Table S5. Atomic composition projected on each atomic orbital type for the MO in Yb doped $CsMnBr_3$ NCs localized on the the Yb ion.

atomic kind	s	p	d	f	g
Cs	0.021328	0.005641	0.005500	-	-
Mn	0.002126	0.000292	0.003116	0.000582	-
Br	0.014030	0.010663	0.005542	-	-
Yb	0.003467	0.001281	0.010993	0.914558	0.000883

Table S6. Atomic composition projected on each atomic orbital type for the LUMO (spin-down) in Yb doped Cs_3MnBr_5 NCs. The MO is localized on the second Yb ion.

atomic kind	s	p	d	f	g
Cs	0.001342	0.002716	0.001417	-	-
Mn	0.000073	0.000324	0.001326	0.000885	-
Br	0.001135	0.170221	0.002162	-	-
Yb	0.000050	0.004023	0.001001	0.809748	0.003579

Table S7. Atomic composition projected on each atomic orbital type for the LUMO+1 (spin-down) in Yb doped Cs_3MnBr_5 NCs. The MO is localized on the second Yb ion.

atomic kind	s	p	d	f	g
Cs	0.001008	0.001856	0.001148	-	-
Mn	0.000150	0.000218	0.002205	0.000948	-
Br	0.000982	0.122471	0.002427	-	-
Yb	0.000392	0.003297	0.001284	0.859343	0.002271
